# Quantitative assessment of the troCarWash™ system for automated laparoscopic camera cleaning

**DOI:** 10.1007/s00464-024-10858-z

**Published:** 2024-05-20

**Authors:** Maaz Ahmed, Ashok Gowda, Fatemeh Alavi Naini, Alexander Le, John Treffalls, Robin Torres, Bryan M. Burt

**Affiliations:** 1 Long School of Medicine, University of Texas Health San Antonio, San Antonio, TX USA; 2 BioTex, Houston, TX USA; 3 Baylor College of Medicine, Houston, TX USA; 4 Paul L. Foster School of Medicine, Texas Tech University Health Sciences Center El Paso, El Paso, TX USA; 5 Division of Thoracic Surgery, David Geffen School of Medicine, University of California, 10833 Le Conte Ave., Room 64-128, Los Angeles, CA 90095-7276 USA

**Keywords:** Minimally invasive surgery, Laparoscopic, Endoscopic, Visualization, Camera clean

## Abstract

**Background:**

Soilage of the surgical endoscope occurs frequently during minimally invasive surgery. The resultant impairment of visualization of the surgical field compromises patient safety, prolongs operative times, and frustrates surgeons. The standard practice for cleaning the surgical camera involves a disruption in the conduct of surgery by completely removing the endoscope from the field, manually cleaning its lens, treating it with a surfactant, and reinserting it into the patient; after which the surgeon resumes the procedure.

**Methods:**

We developed an automated solution for in vivo endoscope cleaning in minimally invasive surgery- a port that detects the position of the endoscope in its distal lumen, and precisely and automatically delivers a pressurized mist of cleaning solution to the lens of the camera. No additions to the scope and minimal user interaction with the port are required. We tested the efficacy of this troCarWash™ device in a porcine model of laparoscopy. Four board-certified general surgeons were instructed to soil and then clean the laparoscope using the device. Representative pre- and post-clean images were exported from the surgical video and clarity was graded (1) digitally by a canny edge detection algorithm, and (2) subjectively by 3 blinded, unbiased observers using a semi-quantitative scale.

**Results:**

We observed statistically significant improvements in clarity by each method and for each surgeon, and we noted significant correlation between digital and subjective scores.

**Conclusion:**

Based on these data, we conclude that the troCarWash™ effectively restored impaired visualization in a large animal model of laparoscopy.

**Supplementary Information:**

The online version contains supplementary material available at 10.1007/s00464-024-10858-z.

Minimally invasive approaches have revolutionized surgery. Minimally invasive surgery (MIS) is performed by placing an endoscopic camera into a body cavity through a small port that traverses the body wall. MIS is plagued by frequent visual impairment of the operative field by soilage of the endoscope that occurs during the conduct of surgery. These soilage events occur from (1) blood and other fluids, (2) tissue debris, (3) particulate smoke generated from energy devices, and (4) fogging from the humidity differential between the body cavity and operating room atmosphere.

Soilage events are common during MIS. The state-of-the-art solution for addressing this problem is a multi-step process in which the surgeon stops operating, removes the camera from the patient, manually cleans the lens of the camera, applies a defogging solution to the lens of the camera, reinserts the scope into the patient, and then continues operating. During robotic surgery, the endoscope must additionally be decoupled and then recoupled to the surgical robot. The frequency of these cumbersome camera cleaning events have been shown to occur approximately 6 times per hour during laparoscopic surgical cases [1]. In other words, surgery is completely interrupted by removing the camera from the patient 6 times per hour. Moreover, it has also been shown that approximately 37% of the conduct of laparoscopic cases is performed with impaired surgical vision [1] because the current method of cleaning the surgical camera is burdensome, time-consuming, and frustrating.

This ex vivo cleaning of the endoscope is a major hurdle in the advancement of surgery that has not been sufficiently addressed. This current practice disrupts surgical workflows, increases time in the operating room, increases cost to the healthcare system, amplifies surgeon frustration, and most importantly, impairs patient safety [1–4]. We set out to address with problem with a novel device—a port that automatically and rapidly cleans the surgical camera in vivo, with minimal effort by the operating surgeon, and with minimal disruption of the surgical procedure. The design of this device resembles to a miniature car wash installed within lumen of the port. Herein, we test the efficacy of this troCarWash™ device to restore surgical visualization in a large animal laparoscopy model.

## Materials and methods

No IRB approval or written consents were necessary for this trial as it did not involve human experimental subjects. An animal research protocol was submitted and approved by Baylor College of Medicine (Protocol Number AN-8145) for the use of a live pig in the final testing of this device.

### Device

**Fig. 1:**
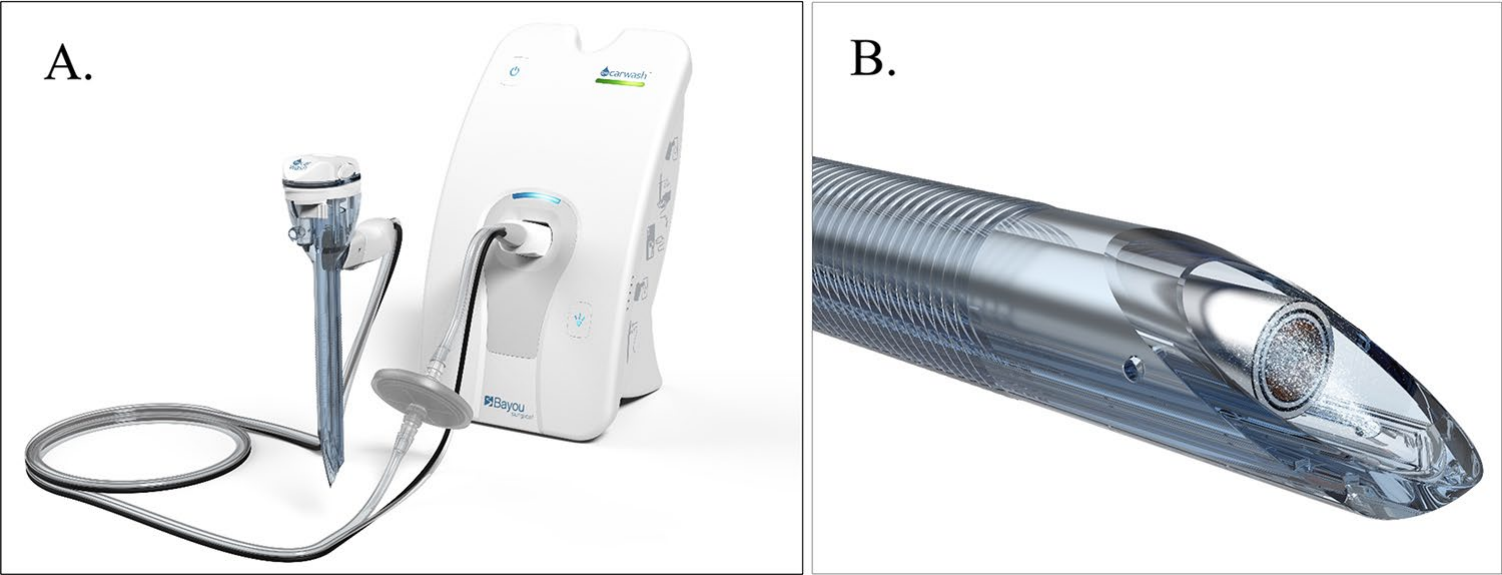
Design of the troCarwash^TM^ device. **A** The device consists of a port, a pump that powers the delivery of a rapid burst of wash fluid, and a set of tubing that connects the port with the pump. **B** Within the port is an optical sensor that detects the light from the scope to identify its location, and a channel through which the pressurized saline wash fluid is directed at the lens of the scope

The troCarWash™ is an FDA-cleared port that cleans the lens of the surgical endoscope within its lumen. It has a 10 mm inner diameter lumen admits 10 mm surgical endoscopes. Within the wall of the port is a small channel that delivers a pressurized burst of 2–3 µl of surfactant containing saline through a small aperture in the distal lumen in the port. Fluid delivery is powered by a pump positioned adjacent to the operating field and which uses the same CO2 source that is used for insufflation. An optical sensor within the wall of the port detects the location of the illuminated surgical endoscope to deliver the misted solution precisely to the lens of the endoscope (Fig. 1). To clean a soiled endoscope, the surgeon simply briefly retracts the tip of the surgical endoscope into the distal end of the trocar where the light from the scope is detected, automatically activating delivery of CO2 and cleaning solution to the lens, and after which the scope is immediately advanced back into the operative field. Total time for the cleaning event is less than 250 ms, and all takes place within the temperate environment of the patient, minimizing conditions favorable for fogging (Video 1).

### Laparoscopy model

A porcine laparoscopy model was used to test the efficacy of this device for restoring surgical visualization. One Yorkshire Cross female pig (35 kg) was anesthetized and intubated by a veterinarian in accordance with an IACUC approved protocol (AN-8145). The troCarWash port was placed by open (Hasson) technique just above the umbilicus and a Storz 10 mm, 30 degree camera was used with a Storz light source. Two 5 mm ports were placed under laparoscopic visualization by standard technique, one in the right upper quadrant and one in the left upper quadrant. A Storz CO2 insufflator maintained 15-mmHg pneumoperitoneum. Four BCM board-certified general surgeons were given a brief instruction on the use of the troCarWash and after a 5-min acclimation period, were asked to perform 10 cycles of soilage and troCarWash cleaning events. Instruction for soilage was not defined and included blood and tissue smudging. The animal was euthanized at the end of the procedure in accordance with the protocol.

### Digital quantification of restoration of image clarity

**Fig. 2:**
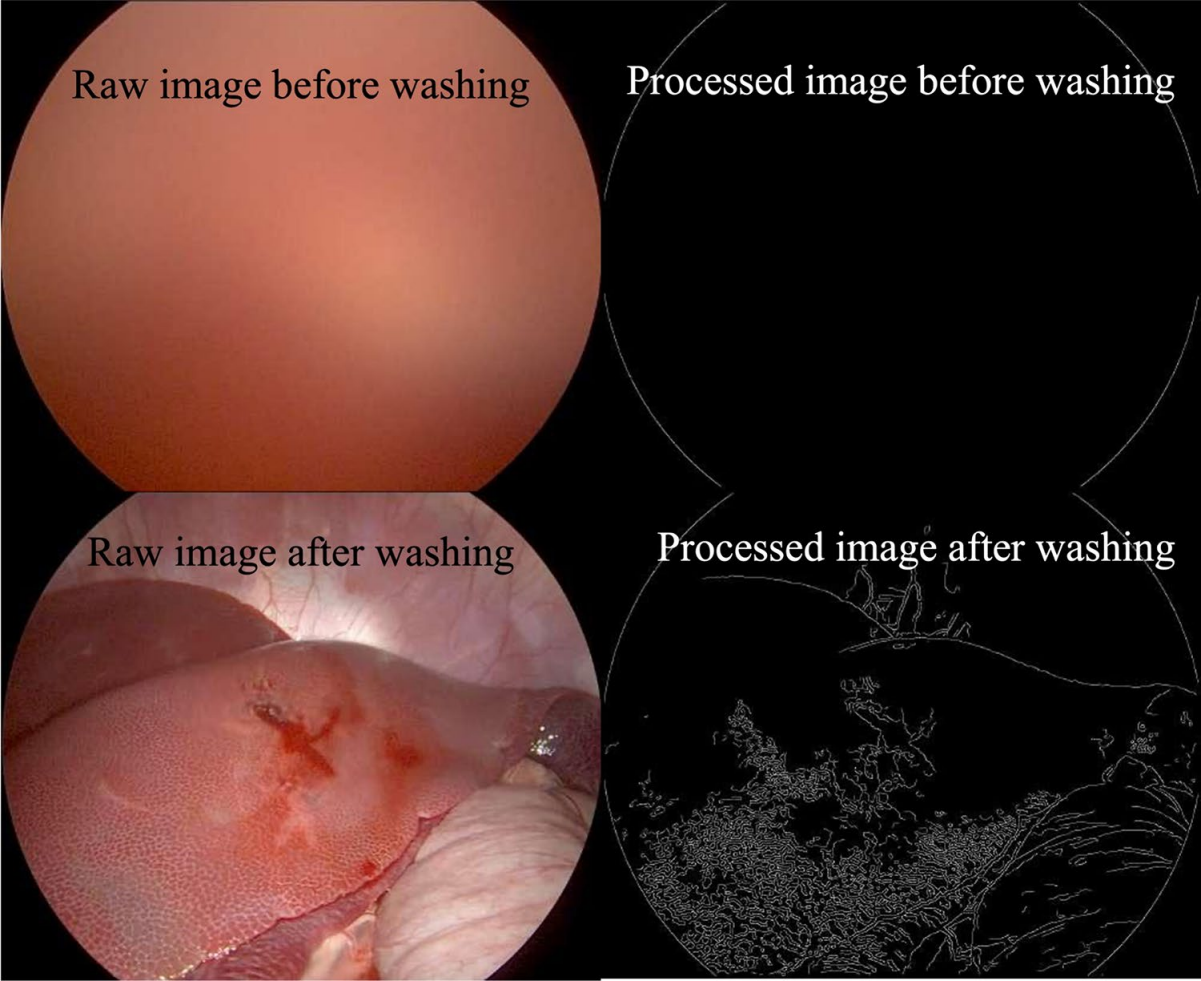
Representation of digital quantification of image clarity. Representative images were obtained from the laparoscopic camera video before and after cleaning with the troCarWash™ (shown on the left). Representative demonstration of the same images processed with Canny edge detector and analyzed by ImageJ to quantify the number of white pixels, which correlate with the number of edges (shown on the right)

Laparoscopic video of the procedure was recorded using Storz equipment and viewed in iMovie software (Apple, Cupertino, CA). For each soilage event, a representative image before cleaning and a representative image after cleaning was exported as a JPEG file for image analysis, by a non-participant in this lab. A canny edge detection algorithm [5] was used to digitally quantify clarity by deconvoluting the edges that naturally demarcate the boundaries of objects and surfaces, which in this case were visceral structures. Binary black-and-white images with single pixel wide edges were generated from the JPEG images using a Canny edge detector to generate images with edges that are just one pixel wide [6]. Mean pixel values were then calculated from the binary images [7] and used as a surrogate for clarity during laparoscopic surgery. Representative examples are shown in Fig. 2.

### Subjective quantification of restoration of image clarity

**Fig. 3:**
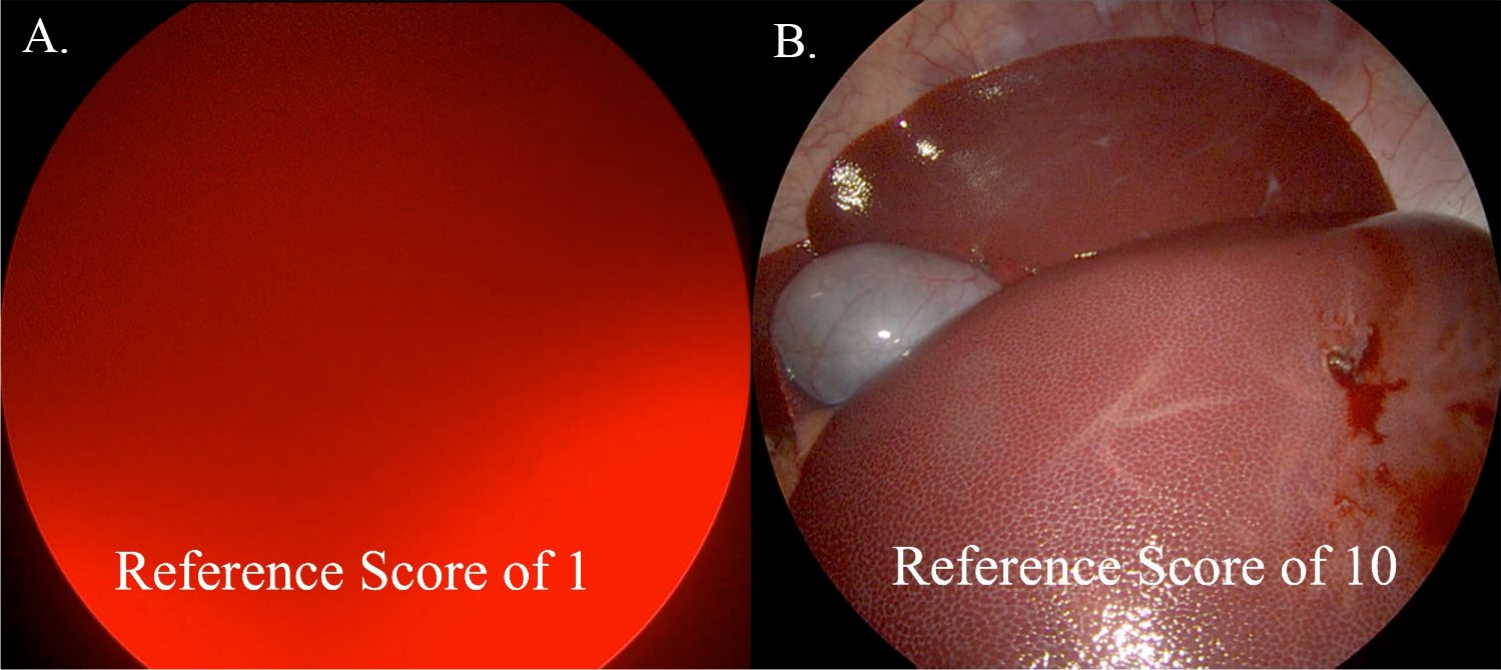
Representation of subjective (observer) quantitation of image clarity. Unbiased, blinded observers were asked to grade clarity of images based a semi-quantitative scale ranging 1 to 10, with 1 being the most unclear (opaque) and 10 being the most clear. Representative images of these extremes are demonstrated

Three medical student observers with no prior information about the experiment were asked to independently score the pre- and post-wash images, which were presented to them in random order. The observers were instructed to use a semi-quantitative scale of 1 through 10, with 1 being the worst quality image (complete obstruction of the surgical field), and 10 being completely clear. Reference images for a score of 1 and for a score of 10 (Fig. 3) were provided to the observers before beginning their scoring.

### Statistical analysis

Statistical analyses were performed using Stata software. p values for the differences in means between before and after wash groups were calculated using paired t tests. Agreement within the group of three student observers and between digital and observers scores were assessed by Pearson’s Correlation Coefficient. The least squares method (regression) was used to evaluate presence of statistically significant trend in measurement-to-measurement variation in post-wash clarity scores to determine the device’s ability to return visualization to baseline levels.

## Results

### Digital quantification of restoration of image clarity

**Fig. 4:**
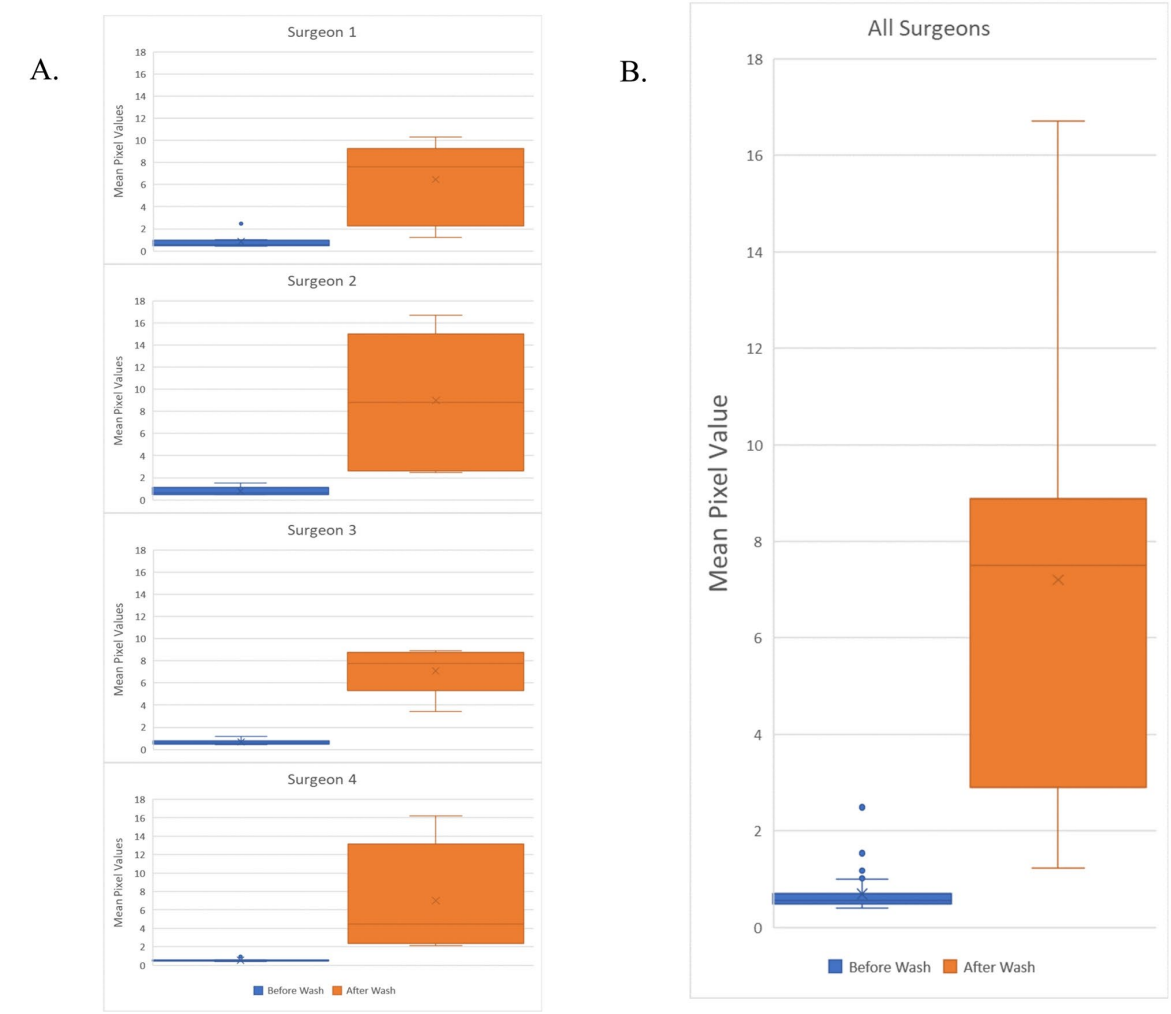
Efficacy of troCarWash™ by digital quantitation of clarity. Mean pixel values of image clarity are shown before and after cleaning with the troCarWash™ for 40 wash events. **A** individual surgeon data and **B** combined surgeon data are shown

A total of 40 wash events were performed and a total of 80 images were generated, with 40 images generated before cleaning and 40 images generated after cleaning. Mean pixel values were higher in post-wash images than pre-wash images (0.690 ± 0.373 versus 7.206 ± 4.242, *P* < 0.001). The differences between before and after-wash mean pixel values were significant when data were stratified by individual surgeons (Surgeon 1: 0.857 ± 0.610 versus 6.467 ± 3.342, *P* < 0.001; Surgeon 2: 0.809 ± 0.373 versus 8.992 ± 5.346, *P* < 0.01; Surgeon 3: 0.672 ± 0.224 versus 7.102 ± 1.706, *P* < 0.001; Surgeon 4: 0.543 ± 0.113 versus 6.996 ± 5.286, *P* < 0.001) (Fig. 4).

### Subjective quantification of restoration of image clarity

**Fig. 5:**
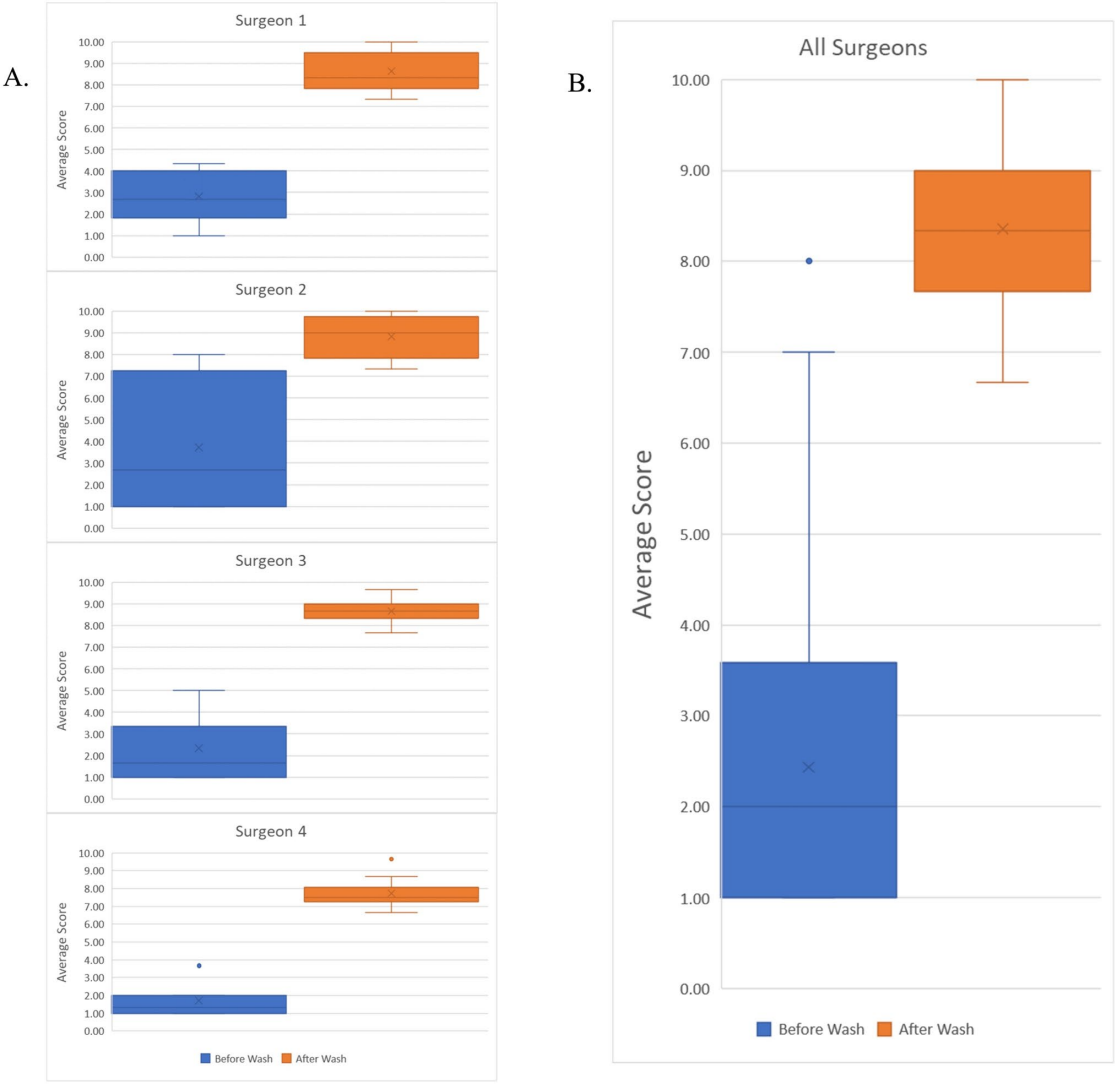
Efficacy of troCarWash™ by subjective (observer) quantitation of clarity. Mean observer scores of image clarity are shown before and after cleaning with the troCarWash™ for 40 wash events. **A** individual surgeon data and **B** combined surgeon data are shown

Comparing the 40 pre-wash and 40 post-wash images, observer mean clarity scores were 2.43 ± 1.68 before cleaning versus 8.36 ± 0.92 after cleaning (*P* < 0.001). Differences remained significant when data were stratified by individual surgeons (Surgeon 1: 2.82 ± 1.16 versus 8.63 ± 0.91, *P* < 0.001; Surgeon 2: 3.72 ± 2.80 versus 8.83 ± 0.92, *P* < 0.001; Surgeon 3: 2.33 ± 1.41 versus 8.67 ± 0.59, *P* < 0.001; Surgeon 4: 1.71 ± 0.90 versus 7.74 ± 0.76, *P* < 0.001) (Fig. 5). Intra-observer correlation was significant in scoring images before (*P* < 0.001) and after washing (*P* < 0.001).

### Correlation between digital and subjective quantitation

**Fig. 6:**
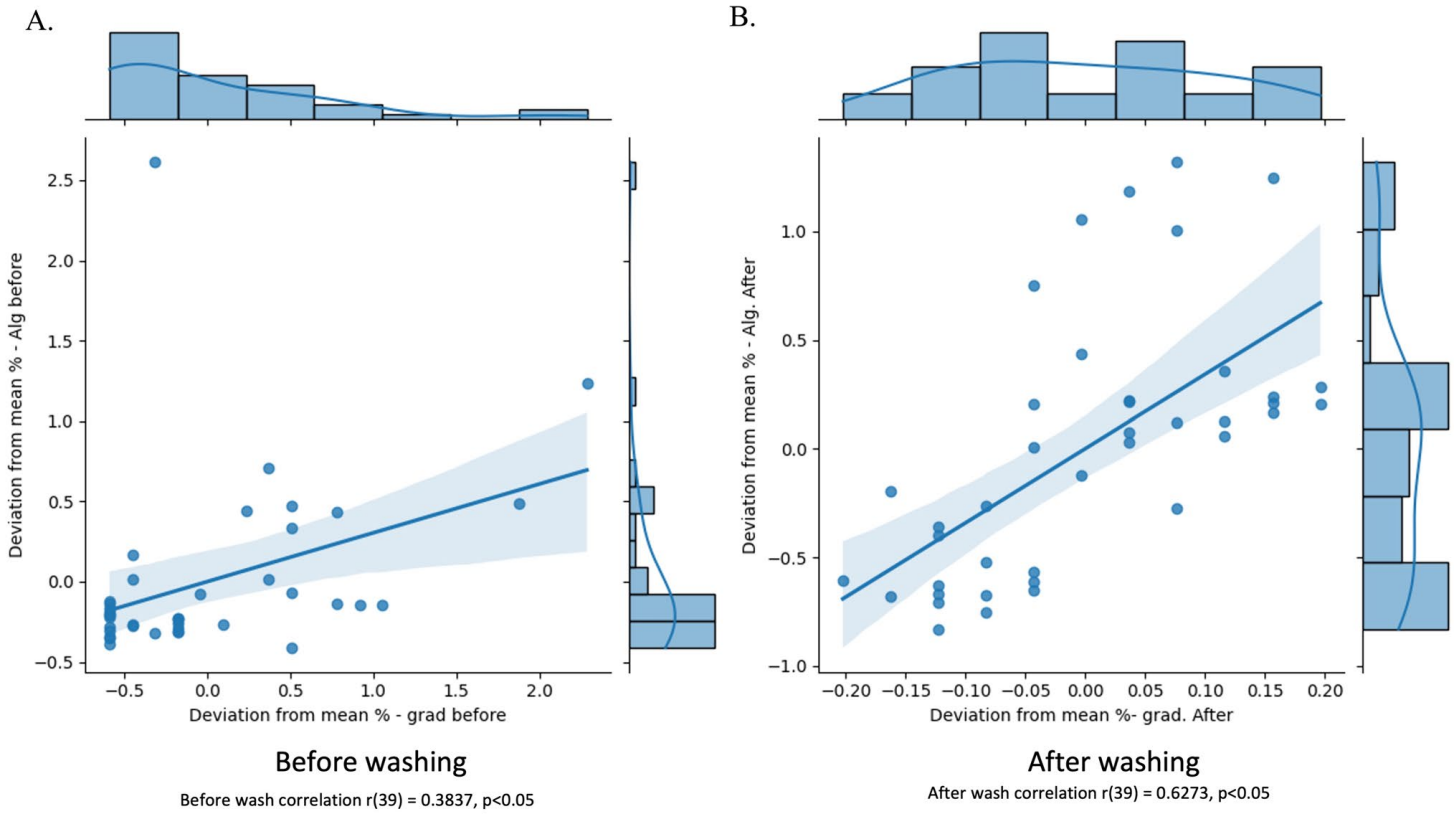
Correlation of between digital and subjective (observer) measure of clarity. For each set of images, pixel values and subjective observer scores were plotted. A confidence interval (blue ribbon) is shown above and below the line of best fit (blue line). **A** Before-wash data and **B** after-wash data are shown. X-axis bars show the distribution of scores by human graders and Y-axis bars the distribution of mean pixel values as determined by our algorithm (Color figure online)

Agreeability between digital and semi-quantitative restoration of clarity was analyzed as the percentage deviation from mean scores. Figure 6 shows positive correlation between observer and digital scores on images before (Pearson’s *r* = 0.38, *P* = 0.015) and after washing (Pearson’s *r* = 0.63, *P* < 0.001).

### Restoration of obstructed visualization to baseline

To determine whether the troCarWash™ could accomplish the same degree of cleaning with repeated use, we plotted each surgeon’s post-wash image quality scores against the surgeon’s wash number. Linear regression was applied to each surgeon’s data to assess trends in worsening image quality with each subsequent wash (which would be represented by a statistically significant negative slope). No statistically significant trend was seen on linear regression for any surgeon between subsequent digital scores (surgeon 1: slope − 0.40, *P* = 0.42; surgeon 2: slope − 1.26, *P* = 0.43; surgeon 3: slope − 0.01, *P* = 0.97; surgeon 4: slope 0.24, *P* = 0.46) or student observer scores (surgeon 1: slope − 0.16, *P* = 0.23; surgeon 2: slope − 0.22, *P* = 0.42; surgeon 3: slope = 0.00, *P* = 0.98; surgeon 4: slope 0.03, *P* = 0.50). These data demonstrate that troCarWash™ remained consistently effective following sequential washing, restoring image quality to pre-soilage levels.

## Discussion

Disruption of surgical workflow has obvious unfavorable implications on the care of patients, and disruptions in the flow of surgery have in fact been shown to result in surgical errors [8]. One of the predominant reasons for such disruption in MIS is unnecessary extracorporeal distraction including the necessary frequent cleaning of the surgical endoscope [9]. Further, the current practice of cleaning the surgical endoscope by completely removing it from the surgical field results in an unavoidable period of complete loss of surgical visualization. Such practice can amplify the severity of acute problems such as the bleeding which may be responsible for the impaired vision. It seems reasonable to submit that a solution for restoring surgical visualization rapidly and without removing the camera from the surgical field, and that maintains better visualization throughout the surgical procedure, could limit the severity of adverse events and improve patient safety.

To this end, we developed a device that maintains surgical visualization by rapid, in vivo, minimal effort cleaning of the frequently soiled endoscope. This animal study was completed prior to the recent FDA clearance of this device and was designed to test its efficacy of cleaning in a relevant large animal model of laparoscopy. In the hands of board-certified general surgeons who had never before seen this device, and with less than 5 min of informal training, our data show that the troCarWash™ effectively and reproducibly restores visualization of the surgical field following common in vivo soilage events.

To increase the rigor of our investigation, we used two modes to evaluate image clarity. First, we used a digital algorithm. We chose the canny edge detection algorithm as the cognitive detection of edges is one of the most basic levels of visual processing used by the brain for higher-level object recognition [10]. Simply speaking, the human brain processes images by determining edges. Second, we used a subjective, semi-quantitative method for grading image clarity, performed by 3 unbiased observers who were unaware of the experimental design and simply asked to score the clarity of laparoscopy images. By each method, and for each surgeon, the troCarWash™ significantly restored visual clarity. The strength of this conclusion is supported further by our finding of statistical correlation between digital deconvolution and semiquantitative observations.

Additional benefits of a rapid, effective, and intuitive use device for in vivo cleaning of the surgical camera includes reducing operating room time, which subjects patients to increased risks related to extended hypoperfusion from anesthesia and surgical site infection [11]. Additionally, operating room time is expensive, and its reduction could consequently reduce burden on the health care system [12]. Further, and not to be understated, is that this problem is highly frustrating to surgeons [1, 3] and other members of the operating room team including learners, assistants, and staff. Although perhaps not rigorously studied, a frustrated operating room can have unfavorable consequences that extend past morale.

Our study was limited to a laparoscopy model with a primary foulant of blood. Whereas we have observed efficacy against other soilage events including adipose tissue, bile, and the particulate smoke from the tissue application of energy devices, they were not included in this initial animal study, though we are planning relevant readouts in prospective human observational studies. For simplicity, we purposely limited this animal study to a 10 mm, 30 ° endoscope, however, our observations from other testing demonstrate excellent efficacy when using 0 ° and 45 °scopes. We have additionally seen efficacy in all brands of scopes that we have tested, and minimal impact of the relationship of the angle of the lens to the device. The device has been cleared by the FDA.

## Conclusion

The troCarWash™ is an automated solution for in vivo endoscope cleaning in MIS and operates without removing the camera from the operative field, without additions to the scope, and with minimal user interaction with the system. The troCarWash™ effectively restores impaired visualization in a large animal model and is promising for use in patients.

### Supplementary Information

Below is the link to the electronic supplementary material.

Supplementary file1 (MP4 107650 KB)
